# Association of Helicobacter Pylori With Development of Peptic Ulcer Disease Among Cirrhotic Patients: An Evidence From Population-Based Study

**DOI:** 10.7759/cureus.19315

**Published:** 2021-11-06

**Authors:** Tsu Jung Yang, Krithika Dhanasekar, Renu Bhandari, Divya Muraleedharan, Swathy S Chirindoth, Harpreet Kaur, Ruchir Goswami, Prakash Maiyani, Maheshkumar Desai, Dharmeshkumar V Moradiya, Hiteshkumar Devani, Achint A Patel

**Affiliations:** 1 Hospital Medicine, MultiCare Good Samaritan Hospital, Puyallup, USA; 2 Internal Medicine, Sterling Medical Center, Sterling Heights, USA; 3 Medicine, Manipal College of Medical Sciences, Kaski, NPL; 4 Internal Medicine, Larkin Community Hospital, South Miami, USA; 5 Internal Medicine, Kannur Medical College, Kannur, IND; 6 Internal Medicine, BronxCare Health System, Bronx, USA; 7 Epidemiology and Public Health, Icahn School of Medicine at Mount Sinai, New York, USA; 8 Internal Medicine, Gold Coast University Hospital, Southport, AUS; 9 Internal Medicine, Hamilton Medical Center, Medical College of Georgia/Augusta University, Augusta, USA; 10 Internal Medicine, St. John of God Murdoch Hospital, Murdoch, AUS; 11 Dental Medicine, University of Pittsburgh School of Dental Medicine, Pittsburgh, USA; 12 Internal Medicine, Oak Hill Hospital, Brooksville, USA

**Keywords:** prevalence study, population based study, live cirrhosis, peptic ulcer disease, helicobacter pylori

## Abstract

Background: *Helicobacter pylori* (*H. pylori*) plays an important role in causing peptic ulcer disease (PUD) in the general population. However, the role of *H. pylori* in cirrhotic patients for causing PUD is obscure. There are various studies evaluating *H. pylori* association with PUD in cirrhotic patients, but the results have been controversial. We sought to analyze the association of *H. pylori* with the development of PUD in cirrhotic patients from the largest United States population-based database.

Methods: We analyzed Nationwide Inpatient Sample (NIS) and Healthcare Cost and Utilization Project (HCUP) data from 2017. Adult hospitalizations due to cirrhosis were identified by previously validated ICD-10-CM codes. PUD and *H. pylori* were identified with the presence of ICD-10-CM codes in primary and secondary diagnosis fields, respectively. We performed weighted analyses using Chi-Square and paired Student’s t-test to compare the groups. Multivariable survey logistic regression was performed to find an association of *H. pylori* with PUD in cirrhotic patients.

Results: Our study showed that the prevalence of *H. pylori* infection was 2.2% in cirrhotic patients with PUD. In regression analysis, *H. pylori* was found to be associated with PUD in cirrhotic patients (OR 15.1; 95% CI: 13.9-16.4; p <0.001) and non-cirrhotic patients (OR 48.8; 95% CI: 47.5-50.1; p <0.001). In the studied population, *H. pylori* was more commonly seen in the age between 50 and 64 years (49.4% vs 44.1%; p <0.0001), male (63.4% vs 59.9%; p <0.0413), African American (16.3% vs 10.6%; p <0.0001), and Hispanic (26.2% vs 14.9%; p <0.0001). *H. pylori* is more likely to be associated with complicated PUD hospitalizations (51.2% vs 44.2%; p <0.0067). Alcoholism and smoking were more common in *H. pylori* group compared to those without (43.6% vs 35.8%; p <0.0001 and 33.7% vs 24.8% p <0.0001, respectively). Factors associated with increased odds of *H. pylori* infection include African American (OR 2.3, 95% CI: 1.5-3.6), Hispanic (OR 2.6, 95% CI: 1.7-4.0), and smoking (OR 1.5, 95% CI: 1.1-2.2).

Conclusion: *H. pylori* are associated with PUD and concurrent cirrhosis, although it is less prevalent than general population. African American, Hispanic, and smoking were independently associated with increased odds of *H. pylori* infection. Further studies are required to better understand the epidemiology and confirm our findings.

## Introduction

Chronic liver disease and its complications are major health problems. There is evidence to suggest higher bleeding complications, delayed healing, and a greater ulcer recurrence rate in patients with liver cirrhosis compared with the general population [1]. In a study by Kamalaporn et al., the prevalence of peptic ulcer disease (PUD) in cirrhotic patients reported by endoscopy screening studies was approximately 5% to 20% when compared with 2% to 4% in the general population [2]. The reasons for the increased ulcer rate in cirrhotic patients are unknown. Oxidative stress that causes gastric tissue damage in cirrhosis may be one of the components causing gastric lesions and hemorrhage [3]. Studies have shown that PUD has been the cause of upper GI bleeding in almost 10% of cirrhotic patients [4]. An episode of upper GI bleeding can actually worsen the prognosis of cirrhotic patients [5]. Eradication therapy may be beneficial because it diminishes the risk of recurrent PUD [6].

*Helicobacter pylori* (*H. pylori*) are a non-invasive gram-negative bacterium that colonizes gastric mucosa and causes PUD [7]. Although several virulence factors of *H. pylori* have been identified but the precise mechanism by which it causes mucosal damage is yet to be understood [3,8]. *H. pylori* play an important role in causing PUD in the general population. However, its role in cirrhosis remains obscure. There are various studies on *H. pylori-*associated with PUD in cirrhotic patients, but the results have been non-conclusive regarding the association of *H. pylori* with PUD in patients with liver cirrhosis [9-11]. Therefore, there is a need for further study in this area. In the present study, we aim to find an association of *H. pylori* with PUD in patients with liver cirrhosis by using the largest nationally representative database.

## Materials and methods

Data sources

We derived a study cohort from the National (Nationwide) Inpatient Sample (NIS) of the Healthcare Cost and Utilization Project (HCUP), Agency for Healthcare Research and Quality (AHRQ). NIS is one of the largest all-payer publicly available databases on inpatient discharges from U.S. hospitals maintained by the AHRQ. The NIS approximates a 20-percent stratified sample of discharges from U.S. community hospitals, excluding rehabilitation, and long-term acute care hospitals, and contains more than 7 million hospitalizations annually [12]. With the established weights in NIS, this data could be weighted to represent the standardized U.S. population and obtain national estimates with high accuracy [13].

The NIS contains data from 1000 hospitals and includes information on about eight million hospitalizations each year. The database represents a random, 20% stratified sample of all inpatient discharges from 46 states (representing >97% of discharges from all the US hospitals). Variable “DISCWT” (Discharge-level weight) is provided by HCUP to generate national estimates [13]. The sample sizes and the proportions presented in this study are the weighted estimates obtained by applying the discharge level weights. Each individual hospitalization in this database is de-identified and maintained as a unique entry with one primary discharge diagnosis.

Study population and design

We used data of 2017 for analysis purposes based on complete data availability during this time period. We restricted analyses to only adult hospitalizations. We identified various diagnoses of interest by using the International Classification of Diseases, 10^th^ Revision, Clinical Modification (ICD-10-CM) diagnosis codes. Hospitalization with Peptic Ulcer Disease (PUD) was identified with the presence of ICD-10-CM codes of K25xx-K28xx in any diagnostic field that is available in the dataset. These codes have been used by previously published articles from administrative databases such as NIS [14]. Similarly, cirrhosis by previously validated ICD-10-CM diagnosis codes K74.60 and *H. pylori* infection by using B96.81 [15].

Definition of variables

We extracted demographics, hospital-level characteristics (geographical region, size, and teaching status), and patient-level characteristics as supplied as part of NIS. We estimated comorbidities using Elixhauser comorbidity software, which is also supplied by HCUP tools and software [16]. Specific concurrent medical conditions and procedures of interest were identified by ICD-10-CM diagnosis and procedure codes. 

End point 

The primary objective of the study was to estimate the association of *H. pylori* to PUD in cirrhotic patients. The secondary objective was to determine the baseline characteristics and factors associated with *H. pylori* infections in patients with PUD and concurrent cirrhosis. 

Statistical analysis

Descriptive statistics were used to compare and present the baseline population. Categorical variables were compared with the chi-square test, and continuous variables were compared with Student's t-test or Wilcoxon rank-sum test based on the distribution of data variables. To estimate the association of *H. pylori* to PUD in cirrhotic patients, we formulated logistic regression models and adjusted them for potential confounders. First, we formulated a regression model by inserting hospitalization due to PUD as an outcome variable and the presence of cirrhosis and *H. pylori* as independent variables. Then we inserted interaction between cirrhosis and *H. pylori* in a regression model in order to estimate the association of *H. pylori* to PUD in cirrhotic patients. In a final regression model to calculate adjusted estimates, we included patient demographics, comorbidities, and other concurrent factors such as NSAIDs, steroids use, alcoholism, and smoking.

We utilized Statistical Analysis System (SAS) 9.3 (SAS Institute, Cary, NC, USA) for all analyses and included designated weight values to produce nationally representative estimates [13]. For regression models, we used survey procedures to account for the inherent survey design of NIS to produce more robust estimates [17]. We considered a two-tailed p-value <0.05 as statistically significant.

## Results

Association of *H. pylori* with PUD in cirrhotic patients

In 2017, there were 30,421,825 adult hospitalizations in the USA, out of which 956,020 had cirrhosis, and 431,825 had PUD present in their diagnoses. Prevalence of *H. pylori* was 2.2% in cirrhotic patients with PUD and 2.8% in non-cirrhotic patients with PUD. In regression analysis, *H. pylori* was associated with PUD in cirrhotic patients (OR 15.1; 95% CI: 13.9-16.4; p <0.001) and non-cirrhotic patients (OR 48.8; 95% CI: 47.5-50.1; p <0.001).

Baseline characteristics of patients with PUD and concurrent cirrhosis by *H. pylori* status

Demographic characteristics of patients admitted due to PUD with underlying cirrhosis and having *H. pylori* were more likely to be 50-64 years old (49.4% vs 44.1%; p <0.0001), male (63.4% vs 59.9%; p <0.0413), African American (16.3% vs 10.6%; p <0.0001), and Hispanic (26.2% vs 14.9%; p <0.0001). *H. pylori* are more likely to be associated with complicated PUD hospitalizations (51.2% vs 44.2%; p <0.0067). Alcoholism and smoking were more common in the *H. pylori* group compared to those without *H. pylori* infection (43.6% vs 35.8%; p <0.0001 and 33.7% vs 24.8% p <0.0001, respectively). Moreover, low median household income (39% vs 31.8%; p <0.0001) and uninsured/self-pay (18.6% vs 10.1%; p <0.0001) were more prevalent in patients with *H. pylori* infection. Other results are depicted in Table 1. 

**Table 1:** Baseline characteristics of patients with PUD and concurrent cirrhosis by H. pylori status †This represents a quartile classification of the estimated median household income of residents in the patient's zip code. These values are derived from zip code-demographic data obtained from Claritas. The quartiles are identified by values of 1 to 4, indicating the poorest to wealthiest populations. Because these estimates are updated annually, the value ranges vary by year. NSAID= Non-steroidal anti-inflammatory drug; PUD= Peptic Ulcer Disease; SE= Standard Error

Patient Characteristics	Without H pylori	With H pylori	p-value
Total Hospitalizations	38490	860	
Type of PUD			0.0067
Uncomplicated	55.8	48.8	
Complicated	44.2	51.2	
Age in years (mean ± SE)	61 (0.2)	59 (1.0)	< .0001
Age in years (median [q1-q3])	60 (52-69)	57 (49-66)	< .0001
Age in years (%)			< .0001
18-34	3.2	1.2	
35-49	14.7	22.1	
50-64	44.1	49.4	
65-79	29.7	18.6	
>=80	8.4	8.7	
Gender (%)			0.0413
Male	59.9	63.4	
Female	40.1	36.6	
Race (%)			< .0001
White	64.0	44.2	
African American	10.6	16.3	
Hispanic	14.9	26.2	
Others	7.5	9.9	
Missing	2.9	3.5	
Comorbidities (%)			
Obesity	15.1	11.1	0.0010
Hypertension	54.7	50.6	0.0154
Diabetes mellitus without complications	12.0	11.6	0.7725
Diabetes mellitus with complications	18.1	16.3	0.1737
Congestive heart failure	14.7	11.6	0.0129
Valvular heart disease	4.7	3.5	0.1051
Chronic pulmonary disease	18.9	15.7	0.0171
Pulmonary circulatory disease	1.1	1.7	0.0939
Peripheral vascular disease	5.9	4.1	0.0267
Paralysis	1.9	2.3	0.3791
Neurological disorders	8.1	4.7	0.0003
Coagulopathy	39.2	34.3	0.0036
Metastatic cancer	4.0	3.5	0.4158
Weight loss	19.6	15.7	0.0042
Renal failure	20.1	13.4	< .0001
Electrolytes disorders	53.1	50.6	0.1384
Hypothyroidism	11.2	6.4	< .0001
Arthritis	2.4	1.2	0.0201
Alcoholism	35.8	43.6	< .0001
Smoking	24.8	33.7	< .0001
Drug abuse	5.8	7.0	0.1476
Long-term drug use (%)			
Aspirin	7.9	9.3	0.1437
Anti-coagulation/platelet therapy	4.7	2.9	0.0141
NSAIDs	2.1	1.2	0.0675
Steroids	1.8	2.3	0.2456
Median house hold income† (%)			< .0001
1^st^ quartile	31.8	39.0	
2^nd^ quartile	25.9	25.0	
3^rd^ quartile	23.3	19.2	
4^th^ quartile	16.8	13.4	
Primary insurance (%)			< .0001
Medicare/Medicaid	69.3	65.1	
Private including HMO	20.4	16.3	
Uninsured/self-pay	10.1	18.6	
Hospital bed size (%)			< .0001
Small	17.6	9.9	
Medium	29.4	27.3	
Large	53.0	62.8	
Hospital Type (%)			< .0001
Rural	5.4	7.0	
Urban non-teaching	22.3	16.3	
Teaching	72.3	76.7	
Hospital region (%)			< .0001
Northeast	16.2	13.4	
Midwest	21.2	22.7	
South	38.8	33.7	
West	23.8	30.2	

Factors associated with *H. pylori* infection in patients with PUD and concurrent cirrhosis

Several predictors associated with *H. pylori* infection among patients with PUD and concurrent cirrhosis are African American (OR 2.3, 95% CI: 1.5-3.6), Hispanic (OR 2.6, 95% CI: 1.7-4.0), and smoking (OR 1.5, 95% CI: 1.1-2.2). The other results are mentioned in Table 2.

**Table 2:** Factors associated with H. pylori infection in patients with PUD and concurrent cirrhosis †This represents a quartile classification of the estimated median household income of residents in the patient's zip code. These values are derived from zip code-demographic data obtained from Claritas. The quartiles are identified by values of 1 to 4, indicating the poorest to wealthiest populations. Because these estimates are updated annually, the value ranges vary by year. NSAID= Non-steroidal anti-inflammatory drug; CI= Confidence Interval; LL= Lower Limit; UL= Upper Limit

Independent variable/Characteristic	Odd Ratio	95% CI (LL)	95% CI (UL)	P value
Age in years				
<50	Reference			
50	0.8	0.5	1.1	0.1847
Gender				
Male	Reference			
Female	1.0	0.7	1.3	0.8202
Race				
White	Reference			
African American	2.3	1.5	3.6	0.0004
Hispanic	2.6	1.7	4.0	< .0001
Others	1.8	1.0	3.2	0.0634
Comorbidities				
Obesity	0.8	0.5	1.3	0.3639
Diabetes mellitus with or without complications	1.0	0.6	1.7	0.8997
Congestive heart failure	0.9	0.5	1.6	0.7661
Chronic pulmonary disease	0.9	0.6	1.4	0.5578
Paralysis	1.2	0.4	3.5	0.6717
Neurological disorders	0.5	0.2	1.1	0.0784
Metastatic cancer	0.7	0.3	1.9	0.5163
Renal failure	0.5	0.3	1.0	0.0328
Arthritis	0.6	0.2	2.4	0.4779
Alcoholism	1.0	0.7	1.5	0.8621
Smoking	1.5	1.1	2.2	0.0205
Drug abuse	1.0	0.5	1.8	0.9312
Long-term drug use				
Aspirin	1.5	0.9	2.7	0.1134
NSAIDs	0.3	0.0	1.8	0.1780
Steroids	1.6	0.6	4.7	0.3477
Median household income†				
1^st^ quartile	Reference			
2^nd^ quartile	0.9	0.6	1.4	0.6515
3^rd^ quartile	0.9	0.6	1.3	0.5293
4^th^ quartile	0.9	0.5	1.5	0.6211
Hospital Type (%)				
Teaching	Reference			
Rural	1.1	0.6	2.1	0.7337
Urban non-teaching	0.6	0.4	1.1	0.0808
Hospital region				
Northeast	Reference			
Midwest	1.4	0.8	2.4	0.2271
South	1.0	0.6	1.7	0.9093
West	1.3	0.8	2.2	0.3317

## Discussion

Several significant findings of the present study include the following: (1) *H. pylori* were significantly associated with PUD in cirrhotic patients, (2) the prevalence of *H. pylori* among patients admitted with PUD and concurrent cirrhosis was lower than the general population, (3) African American, Hispanic, and smoking were significant factors associated with increased odds of *H. pylori* infection. 

The prevalence of *H. pylori* infection varies with different geographic locations around the world. It ranges from 50%-70% in Asian countries to 15.5% in an Australian study of mainly white population [18]. In a meta-analysis by Hooi et al., the pooled *H. pylori* prevalence estimate for the United States (US) general population was 35.6% [19]. Compared to our findings, previous studies have suggested a higher prevalence of *H. pylori* infection in cirrhotic patients compared to the general population. However, the results varied with the method of diagnosis (serological versus endoscopic diagnosis) [20,21]. A meta-analysis by Vergara in 2002 suggested a high prevalence of *H. pylori* infection without PUD in cirrhotic populations [22]. Asymptomatic *H. pylori* infection might contribute to low prevalence in the present study because the majority of *H. pylori* infections could have been underdiagnosed. This postulate is supported by a Greek study of asymptomatic patients with cirrhosis undergoing screening endoscopy. The study did not find an association between *H. pylori* infection and PUD in cirrhotic patients [5]. Congestive gastropathy from portal hypertension in liver cirrhosis resulting in a hostile environment for *H. pylori* might be contributory [23,24]. Furthermore, a study by Kim et al. suggested that the severity of liver cirrhosis is inversely related to the presence of *H. pylori* infection [25].

*H. pylori* are well known to cause PUD, chronic gastritis, gastric cancer, and gastric mucosa-associated lymphoid tissue lymphoma. Their pathogenic role in liver cirrhosis is not exactly clear. Nonetheless, our results suggested that their presence is more likely to be associated with complicated hospitalizations in the studied population (51.2% vs 44.2%; p <0.0067). This finding is contradictory to previous studies, which suggested that *H. pylori* may not play a role in cirrhotic patients with PUD [23,24,26]. Although the prevalence may be low especially in decompensated cirrhotic patients, the infection is more likely to be associated with follicular gastritis and gastric variceal bleeding. Different types of gastritis from *H. pylori* infection, such as atrophic versus follicular types, can explain the inconsistency in our results. Atrophic type causes low gastric acid secretions due to atrophied parietal cells, whereas follicular type causes increased gastric acid secretions by stimulating gastrin secretion from G cells. *H. pylori*-induced follicular gastritis can be considered as an independent risk factor for gastric variceal bleeding, which can result in complicated hospital admissions [27]. However, it is not exactly clear which type is predominant in PUD patients with cirrhosis.

*H. pylori* infection was more common in middle-aged, African American, or Hispanic, male patients compared to those without the infection according to our study. According to our literature search, we did not notice a similar epidemiologic study in the US population that evaluated the association of *H. pylori* infection with PUD in cirrhotic patients. However, a large histo-epidemiological study by Sonnenberg et al. suggested that *H. pylori* infection is more common in males and low socioeconomic populations [28]. A serological study of *H. pylori* in low-income individuals in the southeastern United States by Epplein et al. suggested that African Americans had two- to six-fold increased odds of *H. pylori* seropositivity after adjusting socioeconomic status [29]. In addition, a single-center histopathological study by Nguyen et al. mentioned the highest risk of *H. pylori* infection among African American and Hispanic populations [30]. In line with previous studies, the present study suggested that being African American or Hispanic increases the odds of *H. pylori* infection by 2.3- and 2.6-folds, respectively. Risk factors contributing to *H. pylori* infection in the studied population could have been similar to the general population hence explaining our results. 

It is well known that alcohol and smoking are related to PUD, whereas their relationship to *H. pylori* infection is not exactly clear. In the present study, smoking increases the odds of *H. pylori* infection by one-and-half folds (OR 1.5, 95% CI: 1.1-2.2, p <0.001). This is in contrast to one Chinese and two Japanese studies that reported a negative association between smoking and *H. pylori* infection [31-33]. On the contrary, a study from Northern Ireland reported a positive correlation, whereas other studies reported no association between smoking and *H. pylori* infection [34-36]. The only Asian study that suggested a positive correlation was a singer center Japanese study that included outpatient male smoking patients [37]. The genetics, racial, and lifestyle differences may explain the discrepancy because the majority of our studied population was white and more closely resembled the Northern Ireland study. Unfortunately, our study was not designed to evaluate the difference in those results. Several studies in different parts of the world have mentioned a negative correlation between alcohol and *H. pylori* infection [31,34,38-41]. Alcohol may exert antimicrobial effects on *H. pylori*, inhibit growth by lowering gastric pH, and affect gastric acid secretion, gastric mucosa, and gastric emptying both directly and indirectly thereby creating a hostile environment for *H. pylori* colonization [42-47]. We did not find a statistically significant association between alcohol and *H. pylori* infection in our studied population (OR 1, 95% CI: 0.7-1.5), so conclusions cannot be made. 

Low socioeconomic status is an independent risk factor for *H. pylori* infection, and this probably remains the same for PUD and cirrhotic patients. As mentioned in our results, factors associated with low socioeconomic status such as low median household income, uninsured/self-pay were significantly associated with *H. pylori* infection. Other risk factors include dietary habits and lifestyle behaviors. Underlying medical comorbidities such as obesity, hypertension, congestive heart failure, and chronic pulmonary disease, etc., cannot predict *H. pylori* infection in the studied population. *H. pylori* are supposedly acquired in the early phase of life; hence its prevalence is likely not affected by medical comorbidities [48].

Our study is the first nationwide study looking at *H. pylori* epidemiology in hospitalized patients with PUD and cirrhosis. Since our results relied on accurate coding practices across the nation, the acquired data could have been confounded and will need to be interpreted with caution. We only studied hospitalized patients, so our results will not be applicable to the general outpatient population. We did not evaluate causation and explanation of our findings, owing to the retrospective nature of our study. Despite the limitations, strengths of our study include a large sample size representative of the general US population and the first nationwide study on this particular subject.

## Conclusions

Finally, we concluded that *H. pylori* are associated with PUD and concurrent cirrhosis. However, it is less prevalent than other studies done in the general population. African American, Hispanic, and smoking were significant factors that increased the odds of *H. pylori* infection. Our results suggested that future large-scale studies are required to further understand the epidemiology and confirm our findings.
